# Studies of Peculiar Mg-Containing and Oscillating Bioapatites in Sheep and Horse Teeth

**DOI:** 10.3390/biom11101436

**Published:** 2021-09-30

**Authors:** Andrzej Kuczumow, Jakub Nowak, Renata Chałas, Maja Ptasiewicz, Przemysław Siejak, Maciej Jarzębski

**Affiliations:** 1 ComerLab Dorota Nowak, Radawiec Duży 196, 21-030 Motycz, Poland; andrzej.kuczumow@gmail.com (A.K.); kubit75@gmail.com (J.N.); 2 Department of Oral Medicine, Medical University of Lublin, 20-093 Lublin, Poland; renata.chalas@umlub.pl (R.C.); maja.ptasiewicz@umlub.pl (M.P.); 3 Department of Physics and Biophysics, Poznań University of Life Sciences, 60-637 Poznań, Poland; przemyslaw.siejak@up.poznan.pl

**Keywords:** tooth apatite, electron microprobe, quantification, Mg and C oscillations, new types of bioapatites

## Abstract

New types of biological apatites have been discovered in molar sheep and horse teeth and are divided in two types. In the first and more general type, the release of Mg ions is parallel to the changes in composition of apatite leading to a final stoichiometric ratio of Ca to P ions, going from dentin depth towards the boundary of enamel with air. Inside dentin, another apatite sub-types were discovered with alternating layers of Mg-rich and C-rich apatites. The approximate formal stoichiometric relationships for these peculiar types of bioapatites are suggested. We identified two kinds of ion-exchanges responsible for formation of peculiar apatites. Various combinations of main and minor elements lead to new versions of biological apatites.

## 1. Introduction

Apatites are very interesting and important minerals [1]. They contain ions of several main elements and chemical groups such as Ca^2+^, PO_4_^3−^, Cl^−^, F^−^, OH^−^, but also consist of many minor components. These apparently less important additions sometimes significantly change the features of original minerals. The activity of some cations and anions or more generally–the ion-exchanges among them and the apatite matrix are essential for biological apatites [2]. The influence of Mg was observed a long time ago [3] and in spite of its relatively small and limited concentration, [4] its role is found to be significant. However, there are still controversies about the amount and the way in which Mg is involved in the apatite. Not only the averaged amounts of Mg are important, since Robinson et.al. measured the spatial distribution of Mg in dental enamel [5]. The presence of Mg also lowers the crystallinity, density, hardness and increase the solubility of teeth. The element exerts serious influence on mechanical characterization of material. In total, the crystallographic simulation has proven that the main crystallographic parameters slightly shrink on the substitution of Mg cations, mainly in location Ca(I) [6]. Other authors claim that this substitution is preferred in location Ca(II) [7]. Mg is preferentially located in dentin rather than in enamel and for that reason, it seems to be responsible for the earlier progress of teeth and bone evolution [8].

There are different conceptions concerning the real involvement of Mg in teeth. Together with the idea of its direct presence in crystallographic set of apatites [9], some scientists are inclined towards the opinion that the ions are included in another material, e.g., crystalline as dolomite or amorphous as calcium phosphate (ACP). There is also the idea that Mg is only adsorbed.

Very recently, the occurrence of Mg in rodent enamel has been coupled with amorphous calcium phosphate (ACP), present in the intergranular phase between hydroxyapatite nanowires [10,11]. ACP may play a significant role in decaying or reconstruction of enamel material. Another similar question concerns the presence of Mg ions in the surface layer of bioapatites [12]. It possibly leads to greater surfacial activity of apatite, important in process of bones and teeth repair.

The interrelationship between Mg and other minor inorganic components of biological apatite is another significant aspect of Mg presence in teeth. Such studies started a long time ago for carbonates [13,14]. The combination of refined analytical techniques was applied for a better understanding of both bulk and surface layers of apatites saturated with both magnesium and carbonates [15].

After such observations, artificial syntheses of Mg-including apatites were proposed [16,17,18,19].

It is interesting that some Mg-containing calcium phosphates participate in pathological excretions in humans. For example, whitlockite (Ca, Mg)_9_(PO_4_)_6_, observed in salivary stones and dental calculi, struvite MgNH_4_PO_4_·6H_2_O and newberyite MgHPO_4_*3H_2_O, in urinary stones [20].

Better knowledge of teeth structure of some popular animals–sheep and horse, was the main aim of this contribution. Mg, which is important in human teeth, shows an even more pronounced role in apatites under scrutiny. Where possible, we propose the quantification of results. Such knowledge should bring an understanding of the usefulness of animal apatites for purposes of dentistry. The discoveries from this contribution rather denied this possibility. However, they shed an important light on the possibility of precise steering of syntheses of some biological apatites. Studies can be considered as a preliminary tests, that open research ways for further investigations, especially by the use of synchrotron-based μ-XRD device.

## 2. Results

### 2.1. Primary Peculiarities

#### 2.1.1. Sheep Teeth Studies

**Figure 1:**
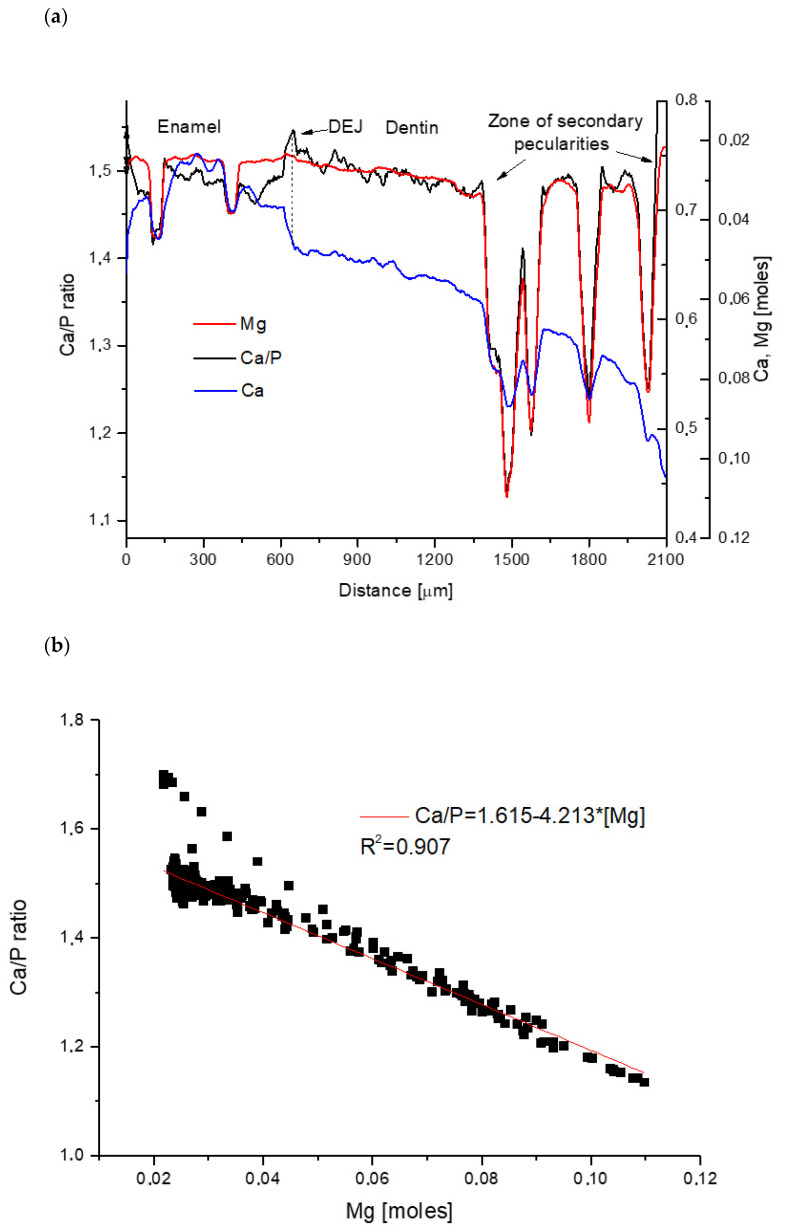

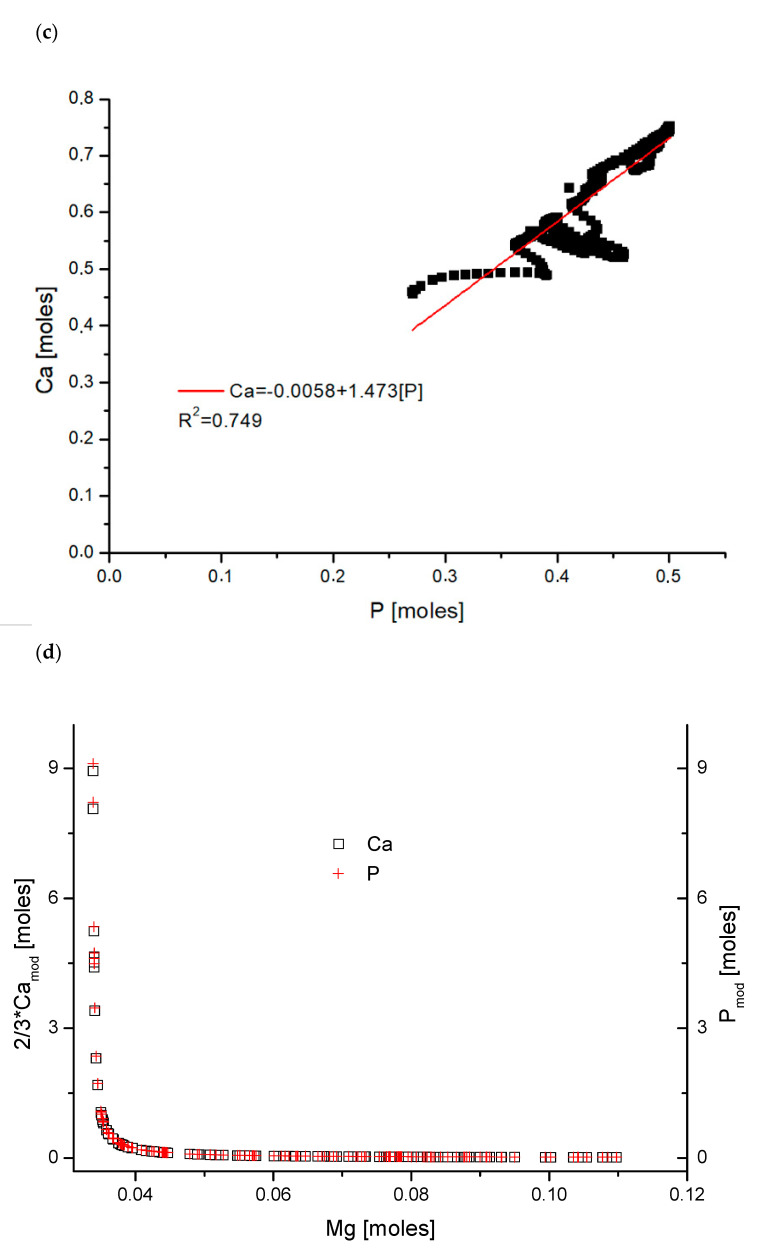
Sheep case: (**a**) strictly convergent spatial profiles of Ca/P and Mg–y axes are inversely oriented; the profile of Ca is added to show the enamel, dentin, and DEJ positions; (**b**) correlation between Ca/P and Mg with relevant equation; (**c**) correlation between Ca and P with equation; (**d**) distribution of Ca(*2/3) and P ions as fine hyperbolic functions of Mg concentration.

The extraordinary convergence of spatial distribution of Ca/P ratio with inverted spatial distribution of Mg contents expressed in moles is shown in Figure 1a. This convergence is better and better while reaching the interior of dentin. It works even if the whole profile is complicated. The complicacy of mutual tailoring of both curves excludes any randomness. This inclines us to correlate both parameters (Figure 1b). The relationship is linear and the ratio increases while Mg contents decrease:

Ca/P = 1.615 − 4.213[Mg](1)

One can observe that slope in this equation is close but not equal to the theoretical value of 1.667.

Since one can logically assume that the contents of Ca and P are strongly positively correlated in apatites, we made the correlation diagram for those two elements (Figure 1c) with the result:

[Ca] = −0.0058 + 1.473[P](2)

This correlation is clear and linear but, surprisingly, somewhat worse than the previous correlation between Ca/P and Mg. The theoretical value of slope connected with [P] variable should be 1.5.

By including Equation (2) into (1), the hyperbolic relationship can be obtained:

[P] = −0.0058/(0.142−4.213[Mg])(3)

Coming back to Equation (1) we can easily deduct concentrations of P and Ca as the functions of Mg contents and finally, the distributions of Ca and P against Mg can be demonstrated as in Figure 1d, where contents of Ca is corrected for the intermolar ratio 2/3, as should be observed in ideal apatites. The figure shows perfect stoichiometric convergence of two main apatite components. These observations suggest that ion exchanges between Ca, P and Mg obey strictly quantitative and possibly stoichiometric rules.

**Figure 2:**
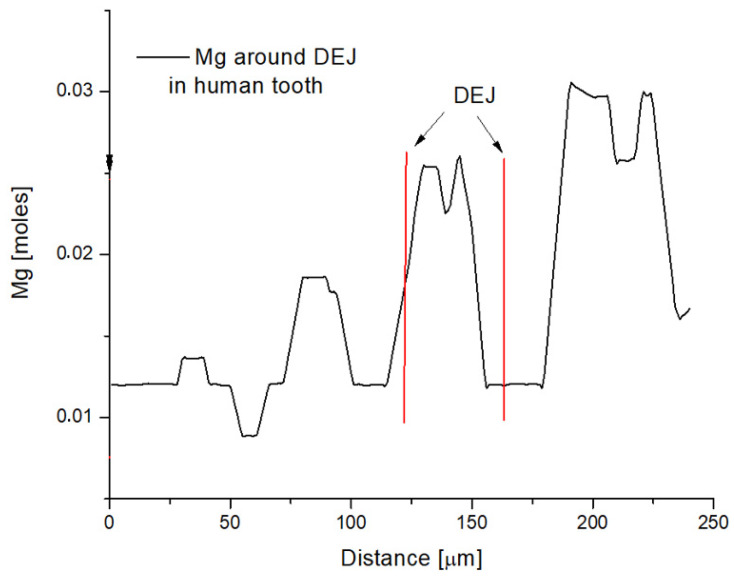
Oscillation of Mg around DEJ in human molar teeth.

The similar oscillations to those ones observed in sheep dentine were discovered inside small fragment of human tooth. Namely, in comprehensive analysis of human dental-enamel and surrounding part of the enamel, a sequence of Mg oscillations was observed in a range of some 200 micrometers inside the boundary enamel and in the middle of the DEJ, as it is shown in Figure 2 [21].

#### 2.1.2. Horse Teeth Studies

**Figure 3:**
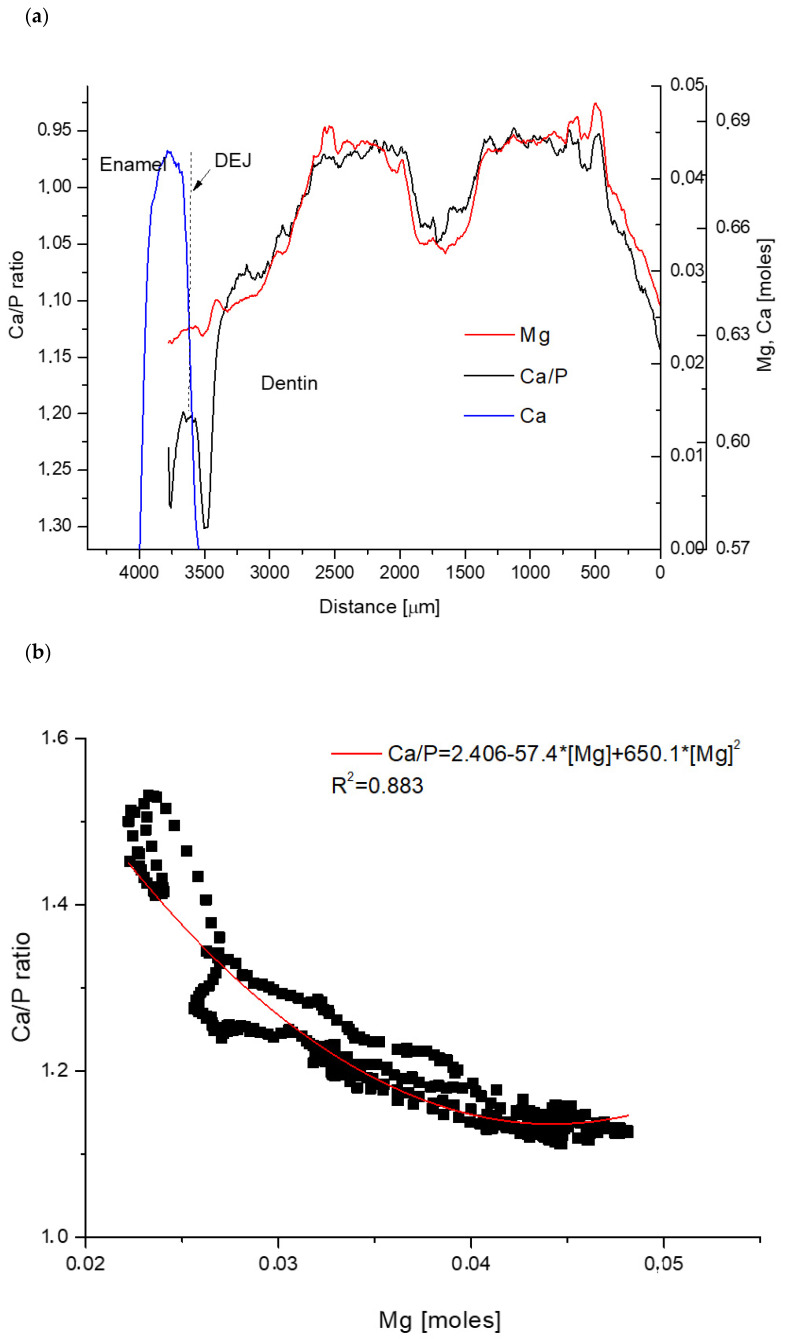

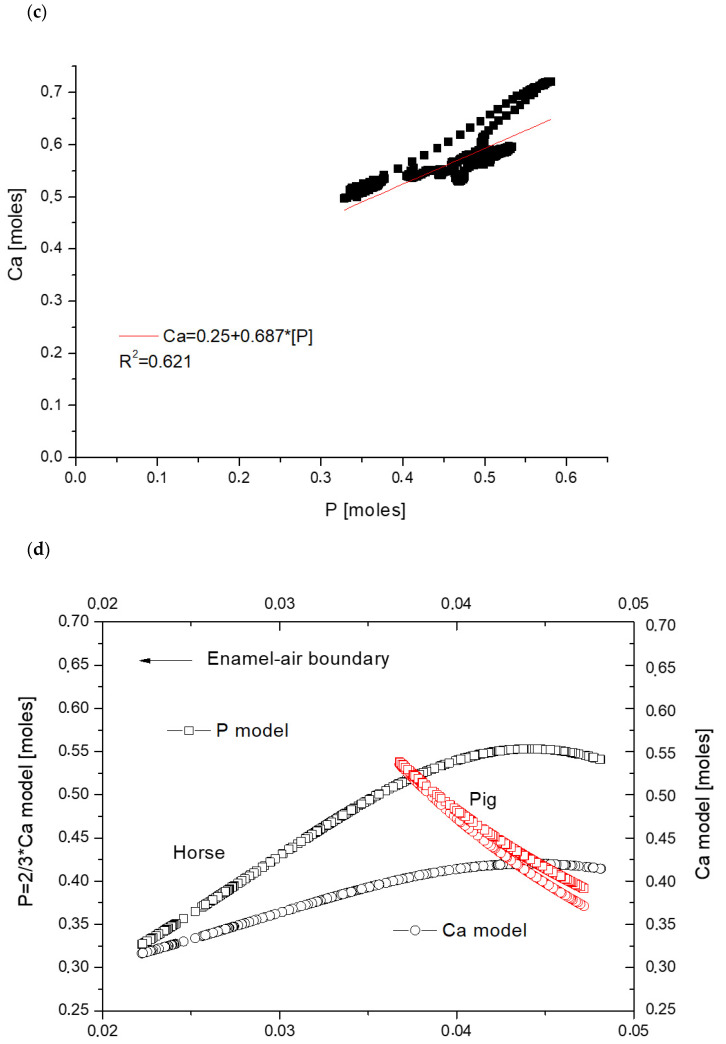

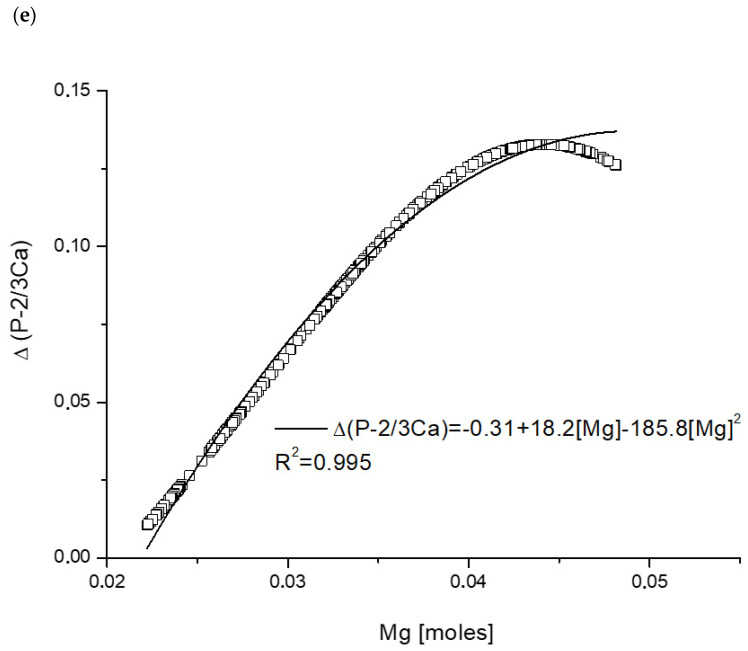
Horse case: (**a**) strictly convergent spatial profiles of inverted Ca/P and Mg; (**b**) correlation between Ca/P and Mg with relevant equation; (**c**) correlation between Ca and P with equation; (**d**) distribution of Ca (*2/3) and P ions as functions of Mg concentration, with addition of similar data for pig; (**e**) variability in the difference of molar amounts of Ca and P as a function of the change in Mg amount.

Figure 3a shows the convergence between the spatial distribution of Ca/P parameter and the spatial profile of Mg ions for horse teeth, which can be transformed in the correlation equation:

Ca/P = 2.406 − 57.4[Mg] + 650.1[Mg]^2^ (4)

This time the relationship is the second order polynomial. The relationship between Ca and P ions, as usually strongly coupled for apatites, is as follows in this case:

[Ca] = 0.25 + 0.687[P](5)

Similarly, as for sheep, this inherent for apatites correlation was weaker than that between Ca/P and Mg.

Then one can derive the hyperbolic relationship:

[P] = 0.25/(1.719 − 57.4[Mg] + 650.1[Mg]^2^) (6)

presented in Figure 3d for both P and Ca. The convergence towards correct stoichiometry in the enamel-air boundary is clear. Here, we cited for comparison our results obtained for porcine teeth, showing similar relationships in the system Ca–P–Mg, described in detail in paper [22]. Figure 3e shows how the release of Mg ions regulates the proportionality of P and Ca ions in apatite. 1 ion of Mg steers the diminishing of the difference between P and 2/3*Ca ions by ~4 ion units. As it was shown in reference [23] for another case, the release of 1 Mg ion moves 9 other ions in the enamel.

One can observe, that the correlation between Ca and P (Figure 3c, Equation (5)) might be stricter if expressed by the polynomials of order higher than 1. However, further equations, analogues of Equation (6), would be incomparably more difficult to solve.

It has been suspected for years that Mg plays some significant, however a little obscure, role in biological apatites. In teeth material studied in this contribution, of sheep and horse origin, one could observe the rigorous parallelism of Ca/P spatial profile and inverted Mg profile. Together with assumption that Ca and P must be relatively strictly coupled in apatites, most probably in a linear manner, it was possible to derive the clearly hyperbolic relationship between Ca or P ions and Mg ion. Assumedly, Mg release from the crystallographic network steers the Ca/P ratio. Going from the interior of dentin towards the boundary of enamel with air, one can observe the final tendency to obtain close to the stoichiometric ratio for Ca and P ions. Having in mind the concept of “overbuilt apatite” [23,24] we have a case Ca/P↑, Mg↓, Cl↑ in direction of boundary enamel-air and one can translate it as the relationships going to idealized formulae:

2Ca_3_(PO_4_)_2_*Ca_2_Mg(PO_4_)_2_*Ca(OH)_2_ → 3Ca_3_(PO_4_)_2_*CaCl_2_ (7)

where the left side means dentin while right one–enamel. Here, the increase in Ca/P can result simply from Mg exchange on Ca and from further consequences of it. We did not pay attention to rigorous stoichiometry, this chemical balance is only formal and educative one. This is the first and essential level of exchanges in apatite under scrutiny. One can simplify above formulae by reducing the recurring terms:

Mg^2+^ + 2OH^−^ → Ca^2+^ + 2Cl^−^ (8)

### 2.2. Second Order Peculiarities

#### 2.2.1. Sheep Teeth Studies

**Figure 4:**
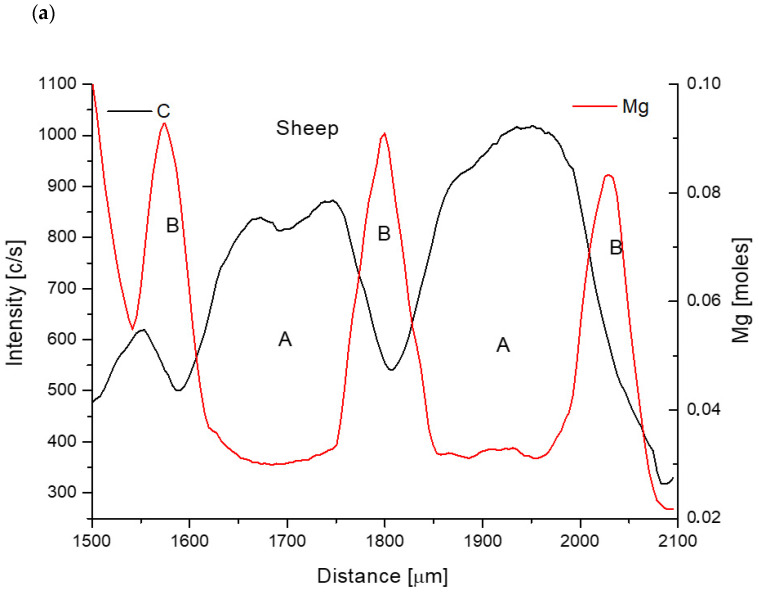

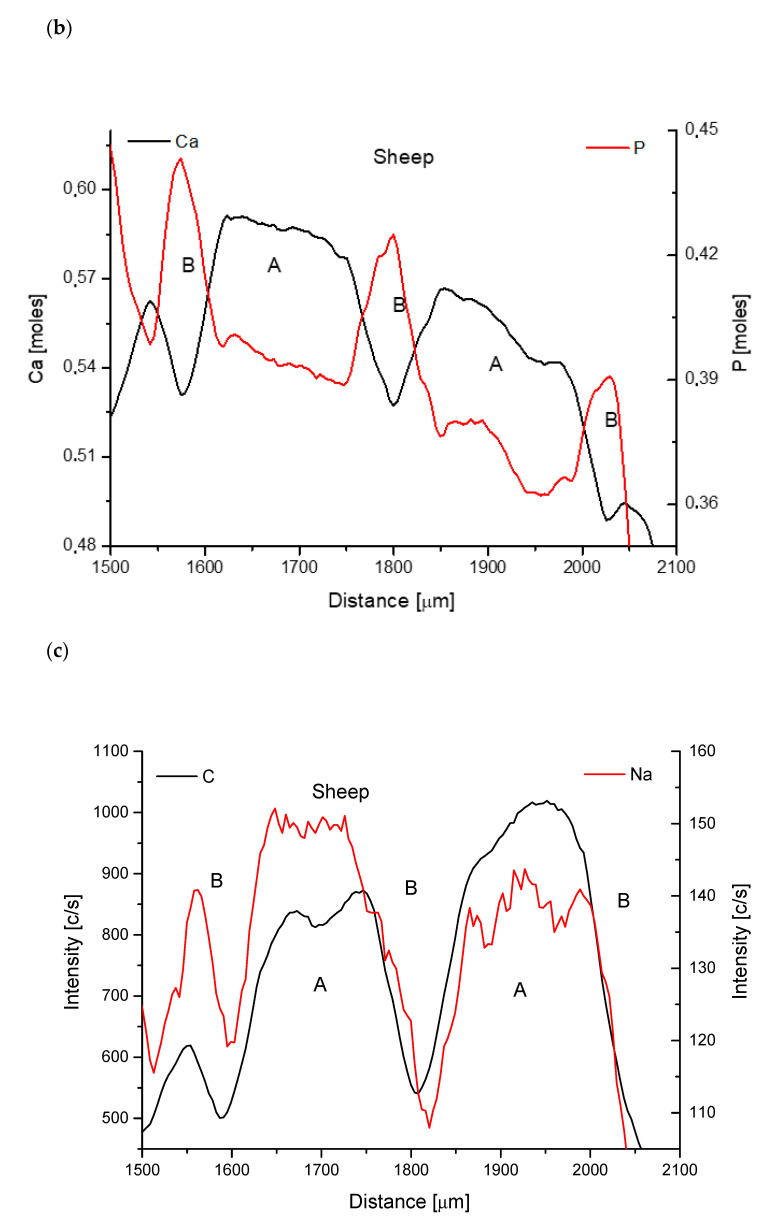
Secondary oscillations of elements in sheep dentin, shown in the pairs: (**a**) Mg-C; (**b**) Ca-P; (**c**) C-Na. Letters a and b’ denote the kind of peculiar apatite in given zone.

Now we must pay some attention to the data from the special area shown in Figure 1a and more specifically in Figure 4a–c. For sheep we have the combination Ca/P↑, Ca↑, P↓, Sr↓, C↑, Na↑, Mg↓, Cl↑, O↓ for growing or falling elemental concentrations in any point (see also Figure 4). The sequence of signals of Ca/P and Mg is the same as in the general case. But the signal of C which is following Ca/P values is a new thing. It means that this time Ca/P is roughly directly proportional to C signal and supposedly to CO_3_^2−^ ion. Those cases correspond to the situation when Mg is released and Ca is going into this location and, in parallel, PO_4_^3−^ ion is substituted by CO_3_^2−^ ion. It is easily explained as passing to ideal situation:

2Ca_3_(PO_4_)_2_*(Ca, Sr, Mg)_3_(PO_4_)_2_*Ca(OH)_2_↔2Ca_3_(PO_4_)_2_*Ca_2_Na(PO_4_) (CO_3_)*Ca(Cl)_2_ (9)

where we have oscillating zones, the first of which (left side of the equation) will be denoted as A, while the second one as B’. We can reduce the formulae by scratching the same terms and neglecting Sr:

Mg^2+^ + Ca^2+^ + PO_4_^3−^ + 2OH^−^
↔ Na^+^ + Ca^2+^ + CO_3_^2−^ + 2Cl^−^ (10)

The sides of equations are located as in Equation (7) above, since we used the combination of arrows, with Ca/P↑ as a reference. The parallel oscillations in concentrations of Ca-P and C–Na can be clearly observed in Figure 4b,c where the variability of Na can be linked to the variability of carbonates. All the oscillations are very clear.

#### 2.2.2. Horse Teeth Studies

**Figure 5:**
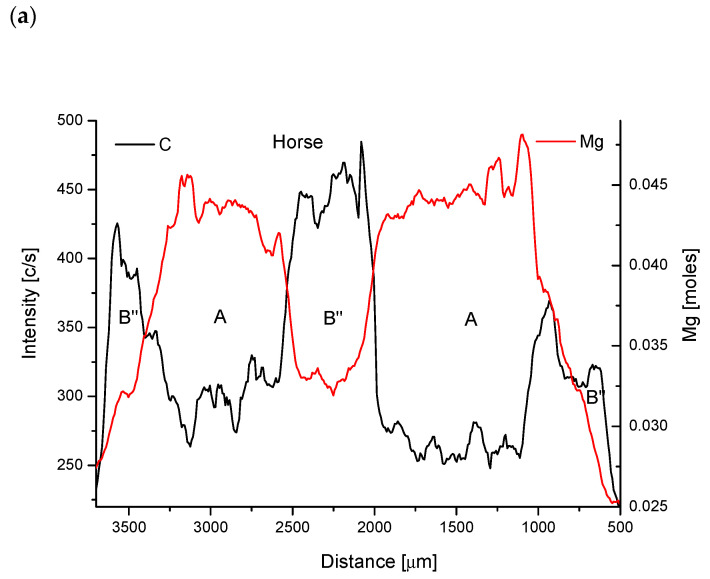

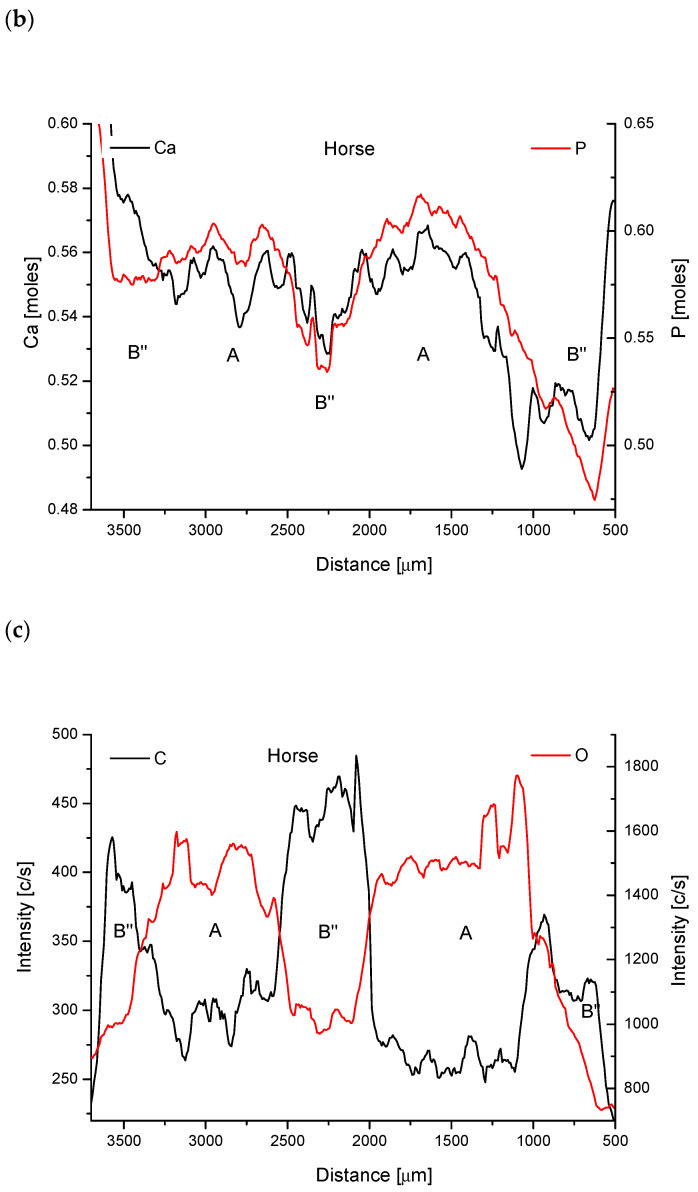
Oscillations of elements in horse tooth, shown in the pairs: (**a**) Mg-C; (**b**) Ca-P, (**c**) C-O. Letters a and b’’ indicate the kind of peculiar apatite in given zone.

Now we must pay some attention to the data from Figure 5a (for horse). They present the situation inside dentin of horse and we see the complimentary oscillation zones for Mg and for C. The relationship between Ca/P and Mg is the same as in the primary case since it covers all the range of teeth and is a universal one for animals mentioned. Secondary combinations can be presented as combination Ca/P↑, Ca↑, P↑, Sr↑, C↓, Na↓, Mg↑, Cl↓, O↑ (not all the data are shown). One must pay attention to the pair Ca-P which behaves in a contrary way in comparison to what was observed for the sheep. It can be presented in idealized form as some kind of “overbuilt apatite”:

2Ca_3_(PO_4_)_2_*CaNa_3_(PO_4_) (CO_3_)* Ca(Cl)_2_↔2Ca_3_(PO_4_)_2_*(Ca,Sr,Mg)_3_(PO_4_)_2_*Ca(OH)_2_ (11)

and is, in fact, similar but inverted side by side as Equation (9). Here, the right side phase is identical as phase A in a chemical equation (Equation (9)), while the left side formula is somewhat different from its counterpart in Equation (9) and will be denoted as B’’. It can be explained by the invocation to the difference in the arrowed combinations of elements mentioned earlier in the text suggesting the greater amounts of Na and smaller of Ca in horse phase B’’. The formula can be reduced as it has been done for sheep:

2Na^+^ + Na^+^ + CO_3_^2−^ + 2Cl^−^
↔ Mg^2+^
+ Ca^2+^ + PO_4_^3−^ + 2OH^−^ (12)

It is an interesting situation since in the world literature, as a rule, the different situation is suggested, when Mg and CO_3_^2−^ ions are acting in one direction. In Figure 5b one can observe going in the same direction, but somewhat more obscure oscillations in concentrations of Ca and P.

## 3. Discussion

Part of variations must result from the simple substitution of Ca in the location of lost Mg, which increases Ca value and substitution of PO_4_^3−^ instead of CO_3_^2−^, resulting in an increased P value. The exchange of CO_3_^2−^ ions (+ Na^+^) on PO_4_^3−^ ions (+ Ca^2+^) is well known as B substitution designated to carbonate ion [25,26]. Hence, since the inverse B substitution demands parallel action of Na^+^ ions, we can imitate B substitution by Na^+^ ions (see Figure 4c).

Next, we have found another peculiar kind of biological apatites, this time occurring only inside the horse and sheep dentins. Here, we observe the oscillation of species expressed by the left and right sides of chemical Equations (9) and (11), respectively. Perhaps, this time the presence of Sr plays a role in developing the oscillations. The convergence towards correct stoichiometry in the enamel-air boundary is clear. We included similar data obtained for porcine teeth [22] in the Figure 3d.

Coming back to the concept of “overbuilt” enamel [23,24] and more generally “overbuilt” apatite, we can postulate the character of variability as expressed by Equation (7) for the whole apatite of sheep and horse teeth. For some of the horse and sheep dentin zones, we have oscillating changes as approximated by Equations (9) and (11). For comparison, the chemical changes in the “overbuilt” phase of human dental enamel are as follows:

3NaCaPO_4_*3CaCO_3_*Mg(OH)_2_ → 3Ca_3_(PO_4_)_2_*CaCl_2_ (13)

and this time the composition of overbuilt enamel is rigorous. This formula can be reduced also:

3Na^+^ + 3CO_3_^2−^ + Mg^2+^ + 2OH^−^
→ 3Ca^2+^ + Ca^2+^
+ 3PO_4_^3−^ + 2Cl^−^ (14)

The concept of “overbuilt” apatite allows for extracting rigorous variations in the apatite composition. One can observe another role of CO_3_^2−^ and Na^+^ pair, that is connected mainly with dentin apatite in human teeth while it plays an even more significant role in the dentin of sheep and horse teeth.

We emphasized by two colors in above reduced formulae two mechanisms of ion-exchanges in peculiar kinds of biological apatites. The first mechanism, denoted in red letters as Mg^2+^ + 2OH^−^ ↔ Ca^2+^ + 2Cl^−^ is more general and concerns the region of hexad axis with nearest surroundings on plane (0,0,1). If it passes to the right, the more ionic structure is formed. The second type of ion exchange is described by the relationship Na^+^ + CO_3_^2−^ ↔ Ca^2+^ + PO_4_^3−^_._ Now, the more swollen structure is formed when the reaction goes on right side. The second ion exchange is known as substitution B for carbonate ion [25,26]. This second type of apatite is superimposed on the first type in different proportions.

It must be considered that the Mg in mouse enamel is observed mainly in the gluing layer concentrated around the apatite rods as the Mg-substituted amorphous calcium phosphate (Mg-ACP) [27,28,29]. although, the amorphous biological apatite is easily transformed into crystalline matter [4,30]. We cannot conclude that the same is true for enamels/whole teeth of other animals. Such a hypothesis would rather suggest that Mg and CO_3_^2−^ ions are included in a nonstoichiometric way and in the external phase [31]. The results of this study shows that ion interchanges are strictly stoichiometric, and we address them to the crystallographic cells. Possibly, the possibility and extent of Mg involvement in apatite must be further explored [32].

Please note that the inverse behaviour of Mg against C in secondary peculiar apatites concerns total contents of carbon, both as CO_3_^2−^ ions and proteinaceous, mainly collagen matter. It testifies that those elements as a whole entities are directly joined in these zones.

Most general view on the peculiar apatites is as follows:

Primary peculiar apatites show following relationships:

Ca/P↑, Mg↓

Secondary peculiar apatites:

Ca/P↑, Ca↑, P↓, Sr↓, C↑, Na↑, Mg↓, Cl↑, O↓

or

Ca/P↑, Ca↑, P↑, Sr↑, C↓, Na↓, Mg↑, Cl↓, O↑,

while normal result for the human enamel shows:

Ca/P↑, Ca↓, P↓, C↑, Na↑, Mg↑, Cl↓,

where the up arrow is always designated to the value Ca/P. This simple analysis indicates that the various combinations of basic and minor elements can lead to numerous versions of biological apatites.

## 4. Materials and Methods

### 4.1. Material Preparation

The sound molar teeth of sheep (4 teeth from different animals) and horse (3 teeth from different animals) were obtained from slaughter-house, during the process of meat production. All the procedures were permitted according to the norms of Polish Animal Welfare Commission.

Animals’ teeth were cut perpendicularly to the main axis of the tooth, with further orientation in buccal-lingual direction. Diamond saw with air cooling was applied for cutting teeth into parts. The cut surface of the middle part of the tooth was polished with the diamond wheel. Polishing was stopped when the roughness was in the range of 1 µm, well acceptable for measurements in SEM. As a final step of preparation, the thin slice was cut off from the rest of tooth, parallel to the prepared smooth surface with a distant of 700 µm. The samples were stored in slightly buffered water (0.1 M of Sorenson P5244 buffer) before measurements. The pieces were next air dried in the air immediately before experiments. To make clear images using backscattered electrons option imaging as well as for electron microprobe scanning (see details p.4.2.), the samples were put with their back, unpolished sides on conductive tape in sample holder chamber. The procedure efficiently avoided charging of bombarded surface.

### 4.2. Chemical Composition Analysis

The scanning electron microscope VEGA LMU manufactured by Tescan (Brno – Kohoutovice, Czech Republic) was coupled with INCA Energy 450 VP attachment together with the energy dispersive detector X-Act Premium (Oxford Instruments, Abingdon, England). The device was used for making elementary chemical analysis, in point analysis and linear scan modes. The diameter of the spot on the sample was ~1 μm, the depth of electron penetration was estimated as ~2.5 μm. The voltage and current were fixed at 25 kV and 0.7 nA, respectively, which was tailored for the optimal analysis of elements between sodium and calcium, which were essential for apatite. The energy resolution of the detector was calculated by the estimation of standard MnKα line. It was equal to 180 eV. The backscattered electron images and optical images of the areas under study were stored in parallel. This provided the possibility of optical scans extraction in locations where chemical measurements were made. The procedure was applied here for sheep and horse teeth and in independent studies for porcine teeth [22].

### 4.3. Quantification

The quantification was performed using PAP procedure [33] with taking pure hydroxyapatite samples (21223 standard from Sigma-Aldrich, Poznań, Poland - now Merck) as a reference sample. Although the difficulties in quantification by EPMA are well known, the procedure was made much easier and justified by very clear systematic trends in spatial profiles. For better observation of stoichiometric relationships, the concentrations of elements and chemical groups were presented in moles. Nevertheless, we were not able to quantify the results for C and O due to the failure of defining moles for elements present in inorganic and organic compounds. The mean values in moles or impulses, where relevant were determined for sheep: Ca 0.63 +/− 0.023 M; P 0.4 +/− 0.012 M; Mg 0.03 +/− 0.0063 M; Na 0.028 +/− 0.007 M; C 603 +/− 18 imp.; for horse: Ca 0.57 +/− 0.02 M; P 0.41 +/− 0.012 M; Mg 0.033 +/− 0.0066 M; C 333 +/− 12 imp; O 1010 +/− 32 imp.

## 5. Conclusions

In conclusion, when analyzing teeth, we found cases in which the Ca/P parameter very rigorously followed the Mg ions spatial distribution. Thorough analysis of this phenomenon allowed for detection of hyperbolic relationships between Ca/Mg and P/Mg concentrations. We strictly determined quantitative relationships between the amount of released Mg and the difference of Ca and P ions. As a result of the process, the stoichiometric ratio of Ca to P aimed for equalization for the edge of enamel, although it did not necessary corresponds to proportions in pure apatite. This mechanism regulated the “perfectness” of apatites.

In the second case, superimposed on the first structure, we found the alternative zones enriched in Mg and C in horse and sheep dentin. This time, Ca, P, Sr, Na, O and CO_3_^2−^ ions participate in ion-exchanges, where the process reveals an oscillatory character. The presence of Mg can be decisive for this process, but it demands further studies. This types of secondary peculiar apatites somewhat differ for sheep and horse teeth.

Two mechanisms of ion-exchanges were revealed in these peculiar types of biological apatites: the first one more general, described as Mg^2+^ + 2OH^−^ ↔ Ca^2+^ + 2Cl^−^ and second one, given in the relationship Na^+^ + CO_3_^2−^ → Ca^2+^ + PO_4_^3−^_._ The second ion exchange is known as substitution B for carbonate ion [25,26].

All the facts testify that we found a very special type of biological apatites in which Mg ions steer the variability of the Ca/P ratio. We suppose that this phenomenon will have a significant application in the controlled synthesis of biological apatites for medical purposes. This possibility results from the identification of two mechanisms of ion-exchange in the materials under scrutiny.

## Data Availability

All data analysis were presented in this manuscript.
